# Association between thyroid hormone levels and hematological indices among women with thyroid disorders

**DOI:** 10.5339/qmj.2025.73

**Published:** 2025-08-12

**Authors:** Sura O. AL-Dewachi, Anmar B. Al-Dewachi, Raad Yahya Alhamdani

**Affiliations:** 1 Department of Pathology, College of Medicine, Ninevah University, Mosul, Iraq; 2 College of Medicine, University of Mosul, Mosul, Iraq *Email: f.medicine@uomosul.edu.iq

**Keywords:** Hypothyroidism, hyperthyroidism, hematological indices, anemia, association

## Abstract

**Background:**

Dysfunctions in thyroid hormones induce various effects on hematopoiesis such as anemia, changes in red blood cells (RBC) and platelet counts, as well as changes in hematological indices. The aim of the present study was to assess the association between thyroid hormone levels and hematological indices among women with thyroid dysfunction who were attending an outpatient clinic in Mosul, Iraq.

**Methods:**

This case–control study was conducted at Al-Wafa Specialist Centre for Diabetes and Endocrine Diseases from April to July 2024. A total of 300 women participated in this study (100 with hypothyroidism, 100 with hyperthyroidism, and 100 with normal thyroid function serving as controls). The hematological indices of the three groups were analyzed and compared. Blood indices were analyzed using the Mythic-18 hematology analyzer. Ethical approval was obtained before data collection, and written consent was obtained from all participants.

**Results:**

All RBC indices [hemoglobin (Hb), RBC count, hematocrit (HCT), mean corpuscular volume (MCV), mean corpuscular hemoglobin (MCH), and mean corpuscular hemoglobin concentration (MCHC)] were significantly lower in patients with hypothyroidism compared to those in the control group (*p* ≤ 0.05), except for red blood cell distribution width (RDW), which was significantly higher in patients with hypothyroidism (*p* = 0.000).

White blood cell count was significantly lower in patient with hypothyroidism compared to those in the control group (*p* = 0.000). In contrast, no statistically significant differences were observed in any blood indices in patients with hyperthyroidism compared to the control group. A statistically significant negative correlation was identified between thyroid-stimulating hormone and Hb, RBC count, HCT, MCV, MCH, alongside a statistically significant positive correlation with RDW.

**Conclusion:**

The dysfunction of thyroid hormones has an impact on blood indices. Anemia is frequently observed in patients with thyroid dysfunction, especially in cases of hypothyroidism. Variations in blood indices may indicate underlying thyroid disorders. Therefore, it is imperative not to disregard the assessment of thyroid hormones in cases of unexplained anemia or unaccounted changes in blood indices.

## 1. INTRODUCTION

Thyroid dysfunction is a leading endocrine disorder that affects approximately 300 million people globally, with an incidence rate ranging between 4 and 10% across various geographical regions.^1^

Thyroid hormones play an integral role in early brain development, skeletal maturation, somatic growth, protein biosynthesis, and hematopoiesis, which includes the synthesis of hemoglobin in adults and its maturation in the fetal stage.^2,3^ During the process of hematopoiesis, thyroid hormones exert a regulatory effect on the cell cycle, as well as the proliferation and differentiation of erythrocytes, leukocytes, and platelets.^4^ Thyroid hormones modulate erythropoiesis by stimulating the production of erythropoietin, as well as promoting the stimulation of erythroid colony-forming units and erythrocyte burst-forming units, which facilitate erythropoiesis.^5,6^ Likewise, thyroid hormones enhance leucopoiesis by enhancing the synthesis of granulocyte–monocyte colony-forming units and interleukin 3.^3,5^ Research indicates that elevated levels of thyroid hormones can reduce the lifespan of thrombocytes, although they are not directly involved in thrombocytopoiesis.^7^

Dysfunction of thyroid hormones (hypothyroidism and hyperthyroidism) significantly influences the production, differentiation, functional capacity, and lifespan of nearly all hematological cells.^7^ Hypothyroidism is often associated with various types of anemia (microcytic hypochromic, normocytic normochromic, and macrocytic normochromic), leucopenia, and thrombocytopenia.^8^ However, anemia is not commonly observed in hyperthyroidism, which may instead present as erythrocytosis and hyperplasia of myeloid lineage. The presence of anemia in hyperthyroidism may result from alterations in iron metabolism, oxidative stress, and hemolysis.^9^ In addition, alterations in various hematological parameters, including mean corpuscular volume (MCV), mean corpuscular hemoglobin (MCH), mean corpuscular hemoglobin concentration (MCHC), and red blood cell distribution width (RDW), are correlated with thyroid dysfunction. Pancytopenia is a rare occurrence, and its underlying etiology remains poorly understood.^10,11^

In developing countries, only a few studies have been conducted to assess the changes in hematological indices related to thyroid dysfunction. Therefore, the aim of the present study was to assess the association between thyroid hormone levels and hematological indices among women with thyroid dysfunction who were attending an outpatient clinic in Mosul, Iraq.

## 2. PATIENTS AND METHODS

This case–control study was conducted at the Al-Wafa Specialist Centre for Diabetes and Endocrine Diseases from April to July 2024. Before starting data collection, preliminary official and ethical approval was obtained from the local health directorate (UOM/COM/MREC/2023-2024/Aug 16), and written consent was acquired from all participants. A total of 300 women were enrolled in this study and subdivided into three distinct groups based on the presence of clinical features indicative of thyroid dysfunction and the results of thyroid-stimulating hormone levels (TSH):

1. Group I (hypothyroid patients): 100 patients who had clinical features of hypothyroidism and TSH levels >5 uIU/ml.
2. Group II (hyperthyroid patients): 100 patients who had clinical features of hyperthyroidism and TSH levels <0.25 uIU/ml.
3. Group III (control group): 100 normal subjects with TSH levels of 0.25–5 uIU/ml.

### 2.1. Inclusion criteria

Inclusion criteria were females aged 20 years and older with a new diagnosis of hypothyroidism or hyperthyroidism.

### 2.2. Exclusion criteria

Exclusion criteria were males, individuals aged <20 years, known cases of hypothyroidism or hyperthyroidism on treatment, individuals with known hematological diseases, anemia, malignancy, bleeding disorders, major trauma or surgery within the past three months, pregnancy, and patients on certain medications such as non-steroidal anti-inflammatory drugs.

The questionnaire used in this study was designed by the author based on previous similar studies^10,11^ and received approval from the scientific committee of the College of Medicine at Mosul University, with a reliability of 91%. Data collection was performed by the authors through direct interviews with the participants.

The questionnaire consisted of three parts:

Part 1: general data regarding the sociodemographic characteristics of the study participants (age, residence, education level, etc.)

Part 2: specific data related to the clinical features of hypothyroidism and hyperthyroidism, history of hematological diseases, history of malignancies, drug history, bleeding disorders, and history of surgery or major trauma.

Part 3: laboratory results (hematological indices and thyroid hormone assay).

From each participant, 5 ml of venous blood were collected using a fully aseptic technique. Furthermore, 2 ml of EDTA anticoagulated blood were used for complete blood count analysis using the “Mythic-18” automatic hematology analyzer,^12^ while 3 ml of whole blood were used for thyroid hormone assay using the Cobas 6000 analyzer.^13^

### 2.3. Statistical analysis

Data analysis was performed using the statistical software program SPSS V23. Results are expressed as frequency and percentage for categorical variables, and as mean ± SD for continuous variables. To assess the statistical differences between the groups, independent t-tests, one-way ANOVA, and chi-square tests were used. A *p* value of ≤0.05 was considered as the cut-off point for statistical significance.

## 3. RESULTS

A total of 300 women participated in this study: 100 with hypothyroidism, 100 with hyperthyroidism, and 100 serving as the control group. No statistically significant differences in general and biochemical characteristics were found between the groups, except for thyroid hormone levels (*p* = 0.000 for all) (Table 1).

Table 2 presents a comparison of hematological indices between hypothyroid patients and those with normal thyroid function. All red blood cell (RBC) indices (hemoglobin (Hb), RBC count, hematocrit (HCT), MCV, MCH, and MCHC) were significantly lower in patients with hypothyroidism compared to the control group (*p* ≤ 0.05), except for RDW, which was significantly higher in patients with hypothyroidism (*p* = 0.000).

In addition, the white blood cell (WBC) count was significantly lower in patients with hypothyroidism compared to those in the control group (*p* = 0.000).

Regarding platelet count, although the mean platelet count was lower in hypothyroid patients compared to the control group, the difference was not statistically significant (*p* = 0.340).

No statistically significant differences were observed in any hematological parameters in patients with hyperthyroidism compared to the control group (Table 3).

Table 4 shows the comparison of hematological parameters between patients with hypothyroidism and hyperthyroidism. The mean values for Hb, RBC, HCT, MCV, MCH, and MCHC were lower in hypothyroid patients compared to hyperthyroid patients, with all differences being statistically significant (*p* ≤ 0.05), except for MCH, where the difference was not statistically significant (*p* = 118). Moreover, RDW was significantly higher in patients with hypothyroidism (*p* = 0.000).

Both WBC count and platelet count were significantly higher in patients with hyperthyroidism compared to those with hypothyroidism (*p* = 0.000 and 0.044, respectively).

The correlation analysis between thyroid hormones (TSH and T4) and hematological parameters is presented in Table 5. A statistically significant negative correlation (*p* ≤ 0.05) was found between TSH and Hb, RBC count, HCT, MCV, and MCH, whereas TSH showed statistically significant positive correlation (*p* ≤ 0.05) with RDW. Moreover, T4 showed a statistically significant positive correlation (*p* ≤ 0.05) with Hb, HCT, MCV, and MCH, along with a negative correlation with RDW.

## 4. DISCUSSION

The thyroid gland plays a pivotal role in the proper development, differentiation, metabolic equilibrium, and normal physiological functions of body organs and tissues, including hematopoiesis. Abnormalities in the secretion of thyroid hormones can lead to a myriad of implications on hematological indices; for example, it may affect RBC, resulting in anemia, or affect WBC and platelets, leading to leukopenia and thrombocytopenia, or it may even cause pancytopenia.^14^

The present study revealed that the levels of Hb, RBC, MCV, MCH, and MCHC were significantly lower in patients with hypothyroidism compared to those in the control group. These findings are consistent with those reported in other studies conducted in India, Iran, Mangolia, and Saudi Arabia.^15–19^ Anemia is a common clinical condition associated with hypothyroidism, which has multifactorial etiologies; these include reduced stimulation of erythroid colonies in the bone marrow by thyroid hormones, reduced oxygen transport to body tissues, reduced erythropoietin production in the absence of thyroid hormone stimulation, and associated deficiencies in vitamin B12 and folate.^20^ Different types of anemia can occur in patients with hypothyroidism (microcytic hypochromic, normocytic normochromic, and macrocytic normochromic).^8^ The present study revealed that normochromic normocytic anemia is the predominant type of anemia, as indicated by the normal MCV in the hypothyroid group, although it is significantly lower than that of healthy controls. Similar results were reported in two other studies conducted in Saudi Arabia, Iraq, and India.^8,11^^,^^15^ Moreover, research conducted in India by Das et al. concluded that normochromic normocytic anemia is the most prevalent type of anemia in patients with hypothyroidism, followed by hypochromic microcytic anemia due to iron insufficiency that may occur during the clinical course of hypothyroidism.^21^

The present study revealed a higher mean RDW in patients with hypothyroidism compared to those with normal thyroid function. This observation may suggest an impact of thyroid hormones on the size of RBC. Similar results were reported in two other studies conducted in India and Turkey.^15,22^

In the present study, the WBC count was found to be significantly lower in the hypothyroid group compared to the control group. Leukopenia is commonly observed in hypothyroidism, which is associated with diverse etiologies, including reduced WBC synthesis and differentiation, destruction of WBC by thyroid-stimulating antibodies, and excessive production of reactive oxygen species, which impairs the integrity of cell surface markers and enhances WBC apoptosis.^23,24^ In addition, platelets have also been assessed in patients with hypothyroidism; while some studies reported no changes in platelet counts in hypothyroid patients,^3^ others showed a reduction in platelet counts in those with hypothyroidism.^15,19,25^

In the present research, all hematological parameters were significantly lower in the hyperthyroid group compared to the control group, except for MCV, WBC, and platelet counts, which were higher in the hyperthyroid group, although the difference was not statistically significant. Anemia is not commonly observed in hyperthyroidism. The occurrence of anemia in patients with hyperthyroidism is attributed to increased body metabolism resulting from excess thyroid hormones, tissue hypoxia, increased erythropoietin production by the kidneys that stimulates erythropoiesis, and an increased demand for iron, B12, and folate. In the present study, the mean Hb level in the hyperthyroid group was normal, a finding that aligns with results from other studies.^9,15^

In cases of hypothyroidism, and regarding WBC and platelets, thyroid hormones have been shown to play a role in the normal production of B cells in the bone marrow by regulating the proliferation of pro-B cells, which may lead to hyperplasia of the myeloid lineage and an increased WBC count.^9,26^ However, thyroid hormones are not directly involved in thrombocytopoiesis, and higher concentration of these hormones may contribute to a reduction in the lifespan of platelets.^7^ In the present study, both WBC and platelet counts were found to be higher in the hyperthyroid group, a finding similar to that reported in other studies.^3^

Regarding the correlation between thyroid hormones and hematological parameters, the present study found a statistically significant negative correlation between TSH and Hb, RBC count, HCT, MCV, and MCH, as well as a statistically significant positive correlation with RDW. Furthermore, T4 showed a statistically significant positive correlation with Hb, HCT, MCV, and MCH, while showing a negative correlation with RDW. These findings are consistent with those reported by other studies conducted in Saudi Arabia and Australia.^27,28^ These significant correlations between thyroid hormones and hematological parameters suggest that T4 and TSH may play a role in the regulation of hematopoiesis, especially erythropoiesis.

It is essential to consider the findings of this research in relation to certain limitations, including hidden confounders that may be present at the time of testing, region of origin, and age-related differences in the levels of thyroid hormones and hematological parameters, as the normal ranges for thyroid hormones can differ from one region to another.^29^ Furthermore, undiagnosed medical conditions may also influence the results.

## 5. CONCLUSION AND RECOMMENDATIONS

In summary, all RBC indices were significantly lower in patients with hypothyroidism compared to those in the control group, except for RDW, which was significantly higher in patients with hypothyroidism. Additionally, the WBC count was significantly lower in patients with hypothyroidism compared to the control group, while no statistically significant differences in any blood indices were found in the hyperthyroidism group compared to the control group. TSH showed a statistically significant negative correlation with Hb, RBC count, HCT, MCV, and MCH, while showing a statistically significant positive correlation with RDW. Changes in hematological parameters may indicate the presence of thyroid disorders, allowing early diagnosis and management. Therefore, it is imperative not to disregard the assessment of thyroid hormones in cases of unexplained anemia in females. Further larger-scale studies are needed to assess the association between thyroid hormone levels and changes in blood indices.

## ETHICAL CONSIDERATIONS

This study was conducted in accordance with the ethical guidelines established in the Declaration of Helsinki. It was performed after obtaining both verbal and analytical consent before data collection. The research protocol, the participant’s information, and consent form were revised and approved by the Medical Research Ethics Committee of the College of Medicine, University of Mosul, as documented under the specific document number (UOM/COM/MREC/2023-2024/Aug 16).

## CONFLICT OF INTEREST

The authors have no conflicts of interest to declare.

## Figures and Tables

**Table 1.** General and biochemical profiles of the study participants.

	Hypothyroidism (*n* = 100)	Hyperthyroidism (*n* = 100)	Controls (*n* = 100)	
Variable	*n* (%)	*n* (%)	*n* (%)	*p*
Age (years)				
20–30	3 (3.0)	6 (6.0)	4 (4.0)	0.982*
31–40	30 (30.0)	28 (28.0)	31 (31.0)	
41–50	33 (33.0)	36 (36.0)	34 (34.0)	
51–60	23 (23.0)	20 (20.0)	19 (19.0)	
60+	11 (11.0)	10 (10.0)	12 (12.0)	
Mean age (years), mean ± SD	45.910 ± 8.574	45.09 ± 10.32	44.920 ± 8.670	0.864**
Residence				
Urban	41 (41.0)	36 (36.0)	39 (39.0)	0.766*
Rural	59 (59.0)	64 (64.0)	61 (61.0)	
Educational level				
Illiterate	23 (23.0)	16 (16.0)	19 (19.0)	0.855*
Primary	39 (39.0)	44 (44.0)	41 (41.0)	
Secondary	24 (24.0)	22 (22.0)	21 (21.0)	
University	14 (14.0)	18 (18.0)	19 (19.0)	
Occupation				
Employed	54 (54.0)	61 (61.0)	58 (58.0)	0.823*
Unemployed	31 (31.0)	29 (29.0)	30 (30.0)	
Retired	15 (15.0)	10 (10.0)	12 (12.0)	
Thyroid hormone level				
Free T3 (pmol/L), mean ± SD	3.917 ± 0.222	6.971 ± 1.310	5.825 ± 1.029	0.000*
Free T4 (pmol/L), mean ± SD	7.596 ± 1.117	26.596 ± 2.659	13.965 ± 1.442	0.000*
TSH (uIU/ml), mean ± SD	16.159 ± 6.412	0.066 ± 0.0399	1.177 ± 0.311	0.000*

*The chi-square test was used to evaluate the differences between groups.

**The one-way ANOVA test was used to evaluate the differences between groups.

**Table 2.** Comparison of hematological indices between hypothyroid patients and the control group.

	Hypothyroidism group (*n* = 100)	Control group (*n* = 100)	*p* *
Parameters	(X ± SD)	(X ± SD)	
Hb (g/dl)	11.53 ± 0.75	13.126 ± 0.857	0.000
RBC (10^6^/μL)	4.69 ± 0.47	5.01 ± 0.73	0.000
HCT (%)	39.23 ± 5.30	41.08 ± 3.03	0.003
MCV (fL)	81.07 ± 1.25	83.33 ± 2.88	0.000
MCH (pg)	26.80 ± 1.30	27.79 ± 2.56	0.001
MCHC (g/dl)	32.49 ± 2.33	33.80 ± 1.18	0.000
RDW (fL)	13.60 ± 0.83	12.86 ± 1.03	0.000
WBC count (10^3^/μL)	6.21 ± 0.975	7.04 ± 0.59	0.000
Platelet count (10^3^/μL)	234.30 ± 62.60	241.80 ± 47.70	0.340

*An independent t-test was used.

Hb: Hemoglobin, RBC: Red blood cells, HCT: Hematocrit, MCV: Mean corpuscular volume, MCH: Mean corpuscular hemoglobin, MCHC: Mean corpuscular hemoglobin concentration, RDW: Red blood cell distribution width, WBC: White blood cells.

**Table 3.** Comparison of hematological parameters between hyperthyroid patients and the control group.

	Hyperthyroidism group (*n* = 100)	Control group (*n* = 100)	*p* *
Parameters	(X ± SD)	(X ± SD)	
Hb (g/dl)	12.96 ± 0.86	13.13 ± 0.86	0.142
RBC (10^6^/μL)	4.89 ± 0.37	5.03 ± 0.73	0.083
HCT (%)	41.42 ± 2.46	41.08 ± 3.03	0.388
MCV (fL)	83.59 ± 3.38	83.33 ± 2.88	0.568
MCH (pg)	27.28 ± 2.75	27.79 ± 2.56	0.178
MCHC (g/dl)	33.64 ± 1.33	33.73 ± 1.19	0.623
RDW (fL)	12.72 ± 0.764	12.86 ± 1.03	0.254
WBC count (10^3^/μL)	7.21 ± 0.49	7.036 ± 0.59	0.843
Platelet count (10^3^/μL)	251.1 ± 55.2	241.8 ± 47.7	0.201

*An independent t-test was used.

Hb: Hemoglobin, RBC: Red blood cells, HCT: Hematocrit, MCV: Mean corpuscular volume, MCH: Mean corpuscular hemoglobin, MCHC: Mean corpuscular hemoglobin concentration, RDW: Red blood cell distribution width, WBC: White blood cells.

**Table 4.** Comparison of hematological parameters between hypothyroid and hyperthyroid patients.

	Hypothyroidism group (*n* = 100)	Hyperthyroidism group (*n* = 100)	*p* *
Parameters	(X ± SD)	(X ± SD)	
Hb (g/dl)	11.53 ± 0.75	12.96 ± 0.86	0.003
RBC (10^6^/μL)	4.70 ± 0.47	4.89 ± 0.37	0.002
HCT (%)	39.23 ± 5.30	41.42 ± 2.46	0.000
MCV (fL)	81.07 ± 1.25	83.59 ± 3.38	0.000
MCH (pg)	26.80 ± 1.30	27.28 ± 2.75	0.118
MCHC (g/dl)	32.49 ± 2.33	33.64 ± 1.33	0.000
RDW (fL)	13.61 ± 0.827	12.72 ± 0.764	0.000
WBC count (10^3^/μL)	6.21 ± 0.97	7.21 ± 0.49	0.000
Platelet count (10^3^/μL)	234.3 ± 62.6	251.1 ± 55.2	0.044

*An independent t-test was used.

Hb: Hemoglobin, RBC: Red blood cells, HCT: Hematocrit, MCV: Mean corpuscular volume, MCH: Mean corpuscular hemoglobin, MCHC: Mean corpuscular hemoglobin concentration, RDW: Red blood cell distribution width, WBC: White blood cells.

**Table 5.** Correlation between hematological indices and thyroid hormones (TSH and T4) among patients with thyroid dysfunction (*n* = 200).

Parameters	Correlation coefficient (r)	
	TSH	T4
Hb (g/dl)	−0.558*	0.562*
RBC (10^6^/μL)	−0.224*	0.132
HCT (%)	−0.269*	0.239*
MCV (fL)	−0.373*	0.408*
MCH (pg)	−0.237*	0.258*
MCHC (g/dl)	−0.093	0.073
RDW (fL)	0.501*	−0.529
WBC (10^3^/μL)	−0.106	0.165
Platelet (10^3^/μL)	0.051	0.139

*A *p* value of ≤0.05 indicates statistical significance.

Hb: Hemoglobin, RBC: Red blood cells, HCT: Hematocrit, MCV: Mean corpuscular volume, MCH: Mean corpuscular hemoglobin, MCHC: Mean corpuscular hemoglobin concentration, RDW:Red blood cell distribution width, WBC: White blood cells.
